# A Ministry of Health for Scotland

**Published:** 1918-09-28

**Authors:** 


### A MINISTRY OF HEALTH FOR SCOTLAND.

The proposed establishment or organisation of a Ministry of Health as part and parcel of the apparatus of Government has necessarily excited discussion in Scotland, even as it has been a subject of debate in the southern part of the kingdom. The existing health authorities in the two countries do not exactly correspond, either in titles or in powers, and naturally there arises inquiry how, both in the one instance and in the other, the present arrangements are likely to be affected by the proposed new departure.

So far as Scotland is concerned, it may be taken for granted that there will be no inclination to accept an inclusion in the English Ministry. Whatever is done, there must be the provision of an independent authority, sensitive to Scottish opinion and in sympathy with Scottish sentiment and customs. The strongest assertion in this direction was advanced at the recent Sanitary Congress in a paper read by Mr. W. M. Marshall, Clerk to the Insurance Committee for the County of Lanark, and from his advocacy, so far as the demand for independent administration is concerned, there is not likely to be much dissent. Mr. Marshall pushes his claim to its logical conclusion. Not only will he not have Scotland controlled by the English Minister, but he insists upon the creation of a new Scottish Ministry having a status and powers practically identical with those to be organised in the southern kingdom. With the device of a Scottish Under-Secretary in the English Ministry he will hold no discussion. Alliance for practical purposes he will welcome, but not subordination. Further, he insists that this same principle of detachment and independence shall be secured for the new Minister in relation also to his Scottish colleagues. He is as opposed to the suggestion that the Secretary for Scotland shall become the Scottish Minister of Health as he is to the proposal that the two countries shall come under one and the same control. Nor will he be satisfied with the position of an Under-Secretary for Health in the Scottish Office. What he demands, in short, is an independent Ministry, and not one merged in the English scheme or controlled by the Scottish Office in Whitehall.

Mr. Marshall is not less clear as to the duties and powers to be attached to the new Ministry. He would transfer to it the functions of the Local Government Board and those of the Insurance Commissioners. Apparently the transference is to be a complete one. Thus it is to include in connection with the Insurance Act not only the supervision of medical benefit, but also that of the payments made in the shape of sickness allowance. The Ministry, in other words, would undertake the work now performed by the National Insurance Commission, and this work, together with the duties now discharged by the Local Government Board, would form the nucleus of the new organisation. Necessarily other duties would be included. The Registrar-General's department would, of course, be one of these, for without accurate statistics neither legislation nor administration could be wisely directed. Mr. Marshall quite rightly lays special emphasis on this point, and the accumulation of facts may perhaps be presented as the first of the duties of any new organisation, by whatever name this may be called. There are impatient people whose haste cannot wait for this necessary foundation, but the half-informed sentimentalist has in his day done harm in many directions, and he is ready to repeat his performance in the department of public health. Stern facts and figures are an essential element in all plans for the future, and a general glow of benevolence is no substitute for them. It is to be hoped that this prosaic consideration will have full weight in the new office, and in his demand for its satisfaction Mr. Marshall will command general sympathy. Whether in his proposal to displace the Scottish Local Government Board by a Scottish Ministry of Health he will also receive general support remains to be seen. The existing position cannot be ignored, nor can it be transformed by a mere stroke of the pen. Everyone agrees that consolidation and co-ordination are desirable qualities, and the existing diffusion of various relations of the public health among a number of different Government departments seems, on the face of it, illogical and absurd. But the reformer has not to write on a clean slate. He has to take things as he finds them, and the Local Government Board in Scotland has many friends ready to testify to the value of the work it has accomplished and is still accomplishing. Possibly the new Ministry might be so arranged as to work through the agency of existing organisations, but the notion that all the present machinery can be scrapped and a brand-new organisation created in its place will hardly be regarded in Scotland as practical politics. What may be the exact arrangements the future alone can tell, but it may be taken as certain that Scotland will insist upon managing its own health affairs, and that it will be concerned less about names than about things. Nor need the English Minister grudge this independence. He will have plenty to do in his own territory. Indeed, if he is to accomplish one-half of what is said to be within his power, his performance will rank high in the catalogue of human achievement.

